# Re: Cosmetic incision for paediatric muscle biopsy

**DOI:** 10.1308/rcsann.2024.0039

**Published:** 2024-07-31

**Authors:** T Burge

**Affiliations:** Salisbury NHS Foundation Trust, UK

COMMENT ON:

**FMT Leone, J Durell, H Dagash and N Patwardhan**. Cosmetic incision for paediatric muscle biopsy. *Ann R Coll Surg Eng*l 2024; **106**: 92.

Sir,

I read the above article with interest.

I was shown much the same technique 20 years ago by Mr Alan Kay, my colleague in the plastic surgery department at Frenchay Hospital, Bristol.

As with the authors, this was designed to avoid the cosmetic problems associated with the biopsies from the thigh or leg that had been used previously.

As well as obtaining a muscle biopsy this approach can also be used to take a skin biopsy (for small nerve fibre studies) and access the lateral cutaneous nerve of the thigh (if a larger nerve is required).

Yours sincerely

